# The Antioxidant and Anti-Inflammatory Activities of 8-Hydroxydaidzein (8-HD) in Activated Macrophage-Like RAW264.7 Cells

**DOI:** 10.3390/ijms19071828

**Published:** 2018-06-21

**Authors:** Eunji Kim, Young-Gyu Kang, Ji Hye Kim, Yong-Jin Kim, Tae Ryong Lee, Jongsung Lee, Donghyun Kim, Jae Youl Cho

**Affiliations:** 1 Department of Genetic Engineering, Sungkyunkwan University, Suwon 16419, Korea; im144069@gmail.com (E.K.); kjhmlkhjml@hanmail.net (J.H.K.); 2 Basic Research & Innovation Division, R&D Center, Amorepacific Corporation, Yongin 17074, Korea; kangyg82@amorepacific.com (Y.-G.K.); jaykim@amorepacific.com (Y.-J.K.); trlee@amorepacific.com (T.R.L.)

**Keywords:** 8-hydroxydaidzein, soybeans, antioxidant, anti-inflammation

## Abstract

8-Hydroxydaidzein (8-HD) is a daidzein metabolite isolated from soybeans. This compound has been studied for its anti-proliferation, depigmentation, and antioxidant activities. However, the anti-inflammatory activities of 8-HD are not well-understood. Through its antioxidant effects in ABTS and DPPH assays, 8-HD reduces the production of sodium nitroprusside (SNP)-induced radical oxygen species (ROS). By triggering various Toll-like receptors (TLRs), 8-HD suppresses the inflammatory mediator nitric oxide (NO) without cytotoxicity. We examined the regulatory mechanism of 8-HD in lipopolysaccharide (LPS)-induced conditions. We found that 8-HD diminishes inflammatory gene expression (e.g., inducible nitric oxide synthase (iNOS), cyclooxygenase (COX)-2, and tumor necrosis factor (TNF)-α) by regulating the transcriptional activities of nuclear factor (NF)-κB and activator protein 1 (AP-1). To find the potential targets of 8-HD, signaling pathways were investigated by immunoblotting analyses. These analyses revealed that 8-HD inhibits the activation of TAK1 and that phosphorylated levels of downstream molecules decrease in sequence. Together, our results demonstrate the antioxidant and anti-inflammatory actions of 8-HD and suggest its potential use in cosmetics or anti-inflammatory drugs.

## 1. Introduction

The human immune system comprises two arms: innate and adaptive immunity. Innate immunity is the first barrier faced by invading pathogens. It recognizes pattern-associated molecular patterns (PAMPs). Adaptive immunity removes pathogens in the late phases of infection and is accompanied by immunological memory [1,2]. PAMPs are recognized by specific pattern-recognition receptors (PRRs). These include the NOD-like receptors (NLRs), RIG-I-like receptors (RLRs), and Toll-like receptors (TLRs). In particular, TLRs recognize a wide range of PAMPs, including lipids, proteins, glycans, and nucleic acids. TLRs therefore serve a pivotal role in inflammation. Each TLR recognizes specific ligands [2,3].

When ligands bind to TLRs, inflammatory signaling pathways are activated via nuclear factor-κB (NF-κB) and activator protein 1 (AP-1) [2,4]. Signals are transduced through adaptor molecules, including myeloid differentiation primary response 88 (MyD88) and TIR domain-containing adaptor-including interferon-β (TRIF). These adaptors regulate the expression of diverse inflammatory genes [4,5,6]. NF-κB signaling induces IκB kinase (IKK) and IκB signal transduction. When signaled by TLRs, IKKs are activated to phosphorylate IκB, which combines with inactive NF-κB [7]. Phosphorylated IκB is degraded by ubiquitination, and free NF-κB translocates to the nucleus to transcribe pro-inflammatory genes, like tumor necrosis factor (TNF)-α, interleukin (IL)-1β, cyclooxygenase (COX)-2, and inducible NOS (iNOS) [8,9,10]. In the AP-1 signaling pathway, signals transduce to IRAK and TAK1 by phosphorylation. These proteins associate with mitogen-activated protein kinases (MAPKs), which are known to be activated by TAK1 [2,10]. Activated MAPKs control the transcriptional activity of AP-1, thereby regulating inflammatory genes, such as MCP-1, IL-1β, and TNF-α [11,12]. These inflammatory molecules play various roles in inflammatory responses.

**Figure 1:**
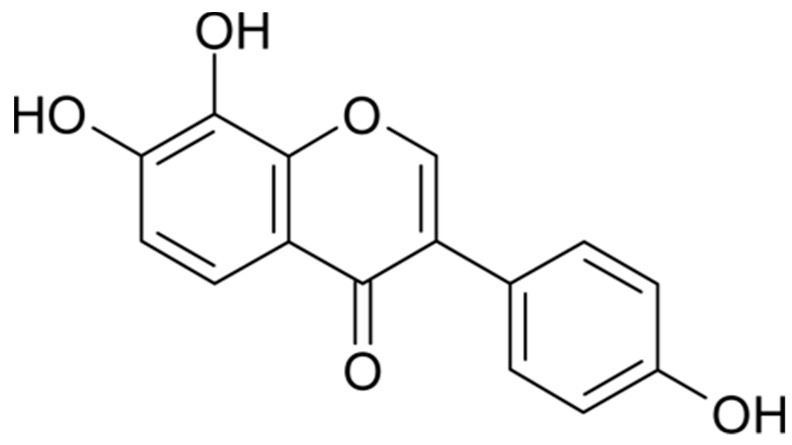
Structure of 8-hydroxydaidzein (8-HD).

Isoflavones are natural diphenolic compounds found in leguminous plants, such as kudzu vine, lupine, and soybean [13,14]. Soybean contains two major isoflavones: daidzein and genistein [15,16,17]. Both daidzein and genistein are biotransformed to monohydroxylated and dihydroxylated metabolites through cytochrome P450-dependent pathways [17]. 8-hydroxydaidzein (8-HD, Figure 1) is one daidzein metabolite isolated from fermented soybeans [18,19]. This compound has recently received attention due to its pharmaceutical and cosmetic effects [14]. Because of its bioactivity and rarity, researchers have sought various methods to produce 8-HD [14,15,20]. Though 8-HD has shown activity relating to anti-proliferation, aldose reductase inhibition, depigmentation, and antioxidation, there have been no reports regarding its anti-inflammatory effects [14,16,19,21]. In this study, we investigated the anti-inflammatory and antioxidant activities of 8-HD and dissected underlying regulatory mechanisms.

## 2. Results

### 2.1. Antioxidant Effects of 8-HD

**Figure 2:**
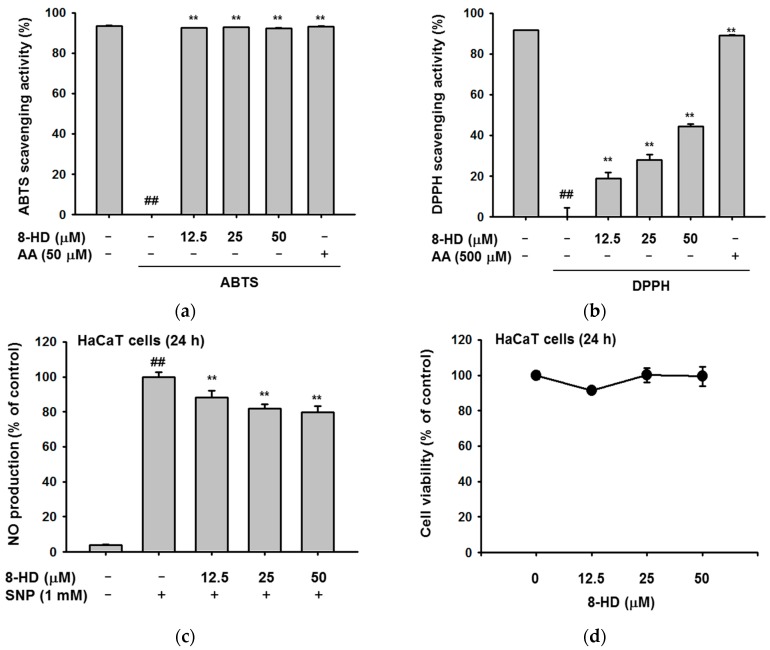
The antioxidant effects of 8-HD. (**a**) 8-HD was incubated with ABTS solution for 30 min at 37 °C. The absorbance was measured by spectrophotometry; (**b**) DPPH and 8-HD were mixed for 30 min at 37 °C. The absorbance at 517 nm was measured; (**c**) RAW264.7 cells were pre-treated with 8-HD for 30 min and then treated with SNP (1 mM) for 24 h. SNP-induced NO production was measured by Griess assays; (**d**) HaCaT cells were treated with 8-HD for 24 h. Cell viability was examined by MTT assays; ## *p* < 0.01 versus a normal group (untreated group); ** *p* < 0.01 versus a control group (induced group); AA: ascorbic acid; SNP: sodium nitroprusside.

We evaluated the antioxidant effects of 8-HD using 2,2′-azino-bis(3-ethylbenzothiazoline-6-sulphonic acid) diammonium salt (ABTS) and 2,2-diphenyl-1-picrylhydrazyl (DPPH) assays. In the ABTS assays, 8-HD cleared ABTS radicals, even at low concentrations (Figure 2a). In the DPPH assays, free radicals were scavenged in a dose-dependent manner (Figure 2b). The IC_50_ values for each experiment were 2.19 μM and 58.93 μM, respectively. Additionally, we treated a keratinocyte cell line (HaCaT cells) with SNP, a NO-releasing reagent. We then examined the effect of 8-HD on NO production. 8-HD slightly decreased the amount of NO without affecting cell viability (Figure 2c,d). Figure 2 shows the antioxidant effects of 8-HD, both in cells and in cell-free systems.

### 2.2. Effect of 8-HD on Nitric Oxide Production

**Figure 3:**
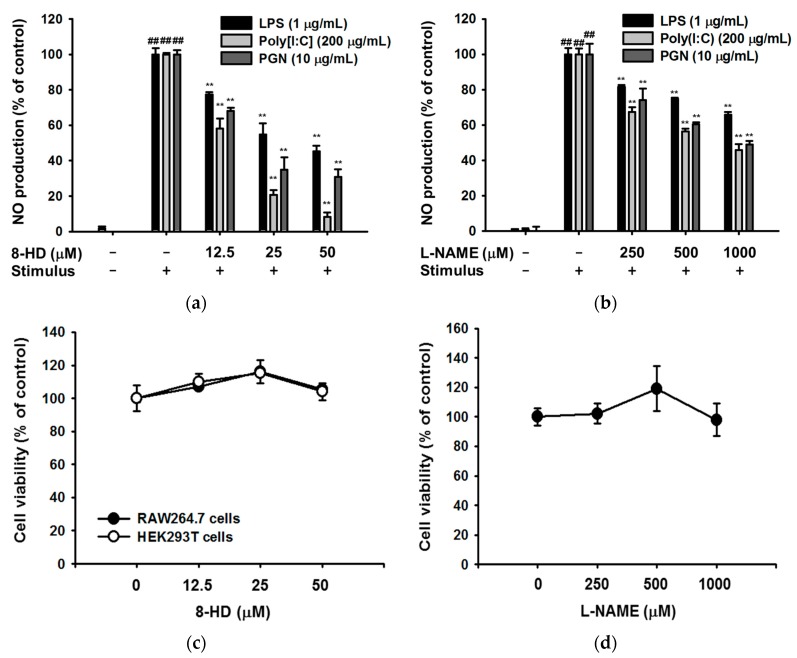
The anti-inflammatory effects of 8-HD. (**a**,**b**) NO production levels for stimulus-treated RAW264.7 cells were determined by Griess assays. RAW264.7 cells were pre-treated with 8-HD (0–50 μM) or L-NAME (0–1000 μM) for 30 min, and cells were then stimulated with Toll-like receptor immunoinducers (lipopolysaccharide (LPS), peptidoglycan (PGN), poly[I:C]) for 24 h; (**c**,**d**) The cytotoxic effects of 8-HD and L-NAME on RAW264.7 and HEK293T cells were tested by MTT assay; ## *p* < 0.01 versus a normal group (untreated group); ** *p* < 0.01 versus a control group (induced group).

Next, we investigated the immunomodulatory effects of 8-HD in a macrophage cell line (RAW264.7 cells). We induced RAW264.7 cells using different stimuli: lipopolysaccharide (LPS, a TLR4 inducer); polyinosinic-polycytidylic acid (poly[I:C], a TLR3 inducer); and peptidoglycan (PGN, a TLR2 inducer). 8-HD suppressed nitric oxide (NO) production without cytotoxicity when cells were stimulated by each inducer (Figure 3a,c). To increase the reliability of the NO assay, we used L-NAME as a positive control. L-NAME also decreased NO production without cytotoxicity (Figure 3b,d). These results suggest that 8-HD suppresses various TLR agonists.

### 2.3. Anti-Inflammatory Effects of 8-HD at the Transcriptional Level

**Figure 4:**
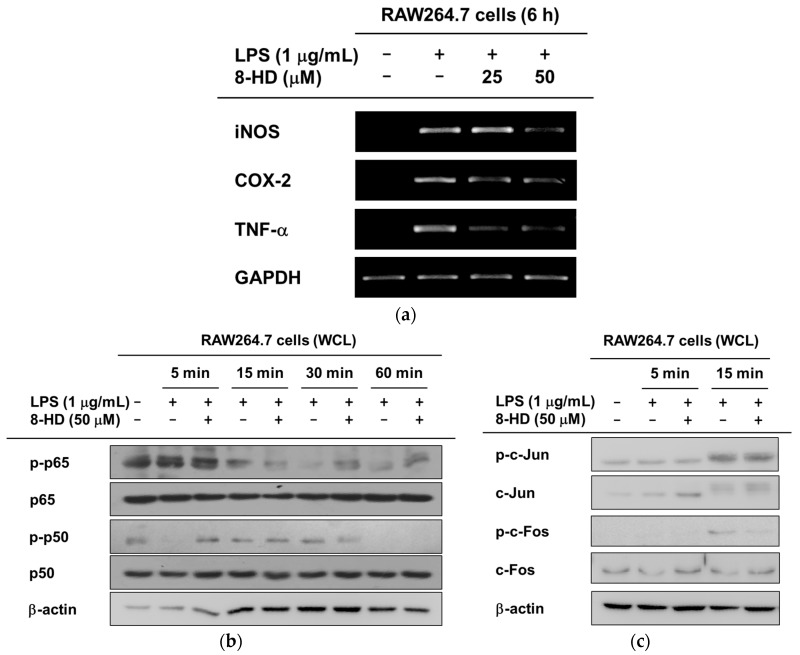
The regulatory mechanism of 8-HD at the transcriptional level. (**a**) The mRNA expression levels of inflammatory molecules were analyzed by semi-quantitative PCR. RAW264.7 cells were pre-treated with 8-HD for 30 min, and LPS was added for 6 h. mRNA was isolated, and RT-PCR was conducted; (**b**,**c**) RAW264.7 cells treated with 8-HD and/or LPS were analyzed in a time-dependent manner (0–60 min). The phosphorylation levels of p65, p50, c-Jun, and c-Fos were determined by immunoblotting. β-actin was detected as a loading control.

Figure 3 shows that 8-HD suppresses the inflammatory mediator production controlled by multiple TLR agonists. We deciphered the regulatory mechanism of 8-HD in controlling TLR4-mediated inflammatory responses. To better understand the regulation of inflammation by 8-HD at the transcriptional level, we isolated mRNA and conducted semi-quantitative PCR to determine the presence of representative pro-inflammatory mediators (iNOS, COX-2, and TNF-α). Under the LPS challenge, 8-HD significantly reduced the expression of iNOS and TNF-α; COX-2 was only slightly affected by 8-HD (Figure 4a). Next, we examined levels of phosphorylated, inflammation-related transcription factors over time. Phosphorylated transcription factors translocate to the nucleus [22,23,24]. Figure 4b shows that phosphorylation of the NF-κB subunits (p65 and p50) was regulated by 8-HD at 15 and 30 min, respectively. Regarding the AP-1 transcription factor, it was found that 8-HD can decrease the phosphorylation level of c-Fos but not c-Jun at 15 min (Figure 4c). These results suggest that 8-HD can regulate inflammation by suppressing the transcriptional activities of NF-κB and AP-1.

### 2.4. Anti-Inflammatory Effects of 8-HD on NF-κB and AP-1 Signaling

**Figure 5:**
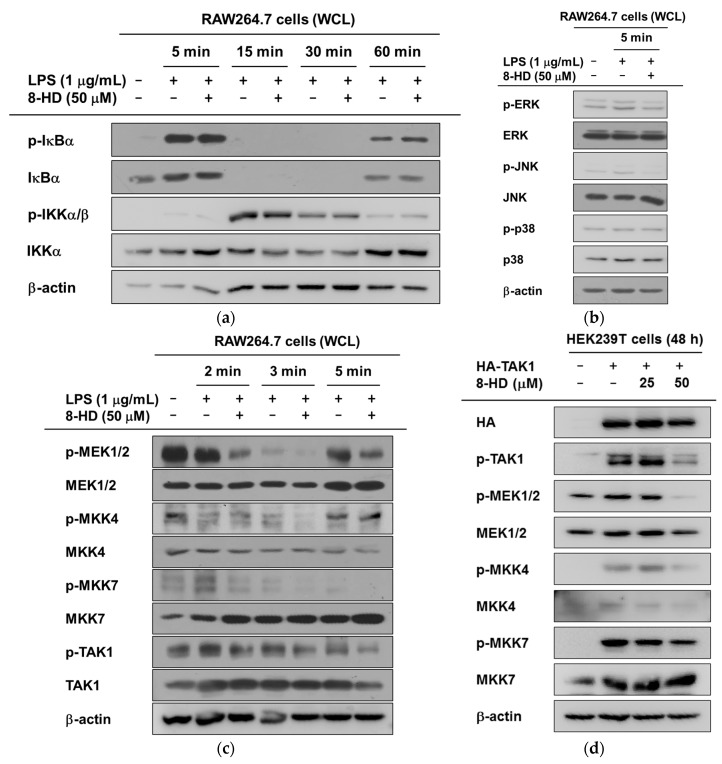
The effects of 8-HD on the NF-κB and AP-1 signaling pathways. (**a**) The effect of 8-HD on the NF-κB signaling pathway using LPS-treated RAW264.7 cell lysates. The phosphorylated forms of NF-κB inhibitory protein (IκBα) and IKKα/β were determined by immunoblotting; (**b**) The effect of 8-HD on the activator protein 1 (AP-1) signaling pathway using whole cell lysates. The phosphorylated levels of the mitogen-activated protein kinases (MAPKs; ERK, JNK, and p38) were measured by immunoblotting; (**c**) RAW264.7 cells were pre-treated with 8-HD for 30 min, and LPS was then applied for different time points (2, 3, and 5 min). The phosphorylated levels of MAPK/ERK kinase 1/2 (MEK1/2), mitogen-activated protein kinase kinase 4 (MKK4), MKK7, and transforming growth factor beta-activated kinase 1 (TAK1) were determined by immunoblotting; (**d**) HA-TAK1 was transfected into HEK293T cells for 24 h. Cells were then incubated for 24 h in the presence or absence of 8-HD. Whole cell lysates were prepared, and immunoblotting was performed. The phosphorylation levels of TAK1, MEK1/2, MKK4, and MKK7 were verified. β-actin was detected as a loading control.

To investigate the way in which 8-HD inhibits NF-κB and AP-1 transcriptional activities, various signaling pathways were analyzed by immunoblotting. First, we assessed the NF-κB signaling molecules IκBα and IKKα/β, which are important for the nuclear translocation of NF-κB via the degradation of IκBα. Interestingly, the NF-κB inhibitory protein IκBα was not regulated by 8-HD (Figure 5a). This result indicates that 8-HD is not able to suppress the transcriptional activities of NF-κB (Figure 4b and Figure 5a), due the absence of IκBα degradation. For the AP-1 signaling pathway, however, 8-HD inhibited the activation of ERK and JNK at 5 min (Figure 5b). We therefore analyzed certain upstream molecules of ERK and JNK at earlier time points. The phosphorylated form of MEK1/2 was clearly decreased at 2 and 3 min. 8-HD blocked the activity of MKK4 at 3 min, and also blocked the activity of MKK7 at 2, 3, and 5 min. The activity of TAK1, a common upstream molecule of the MAPKKs, was also inhibited by 8-HD (Figure 5c).

**Figure 6:**
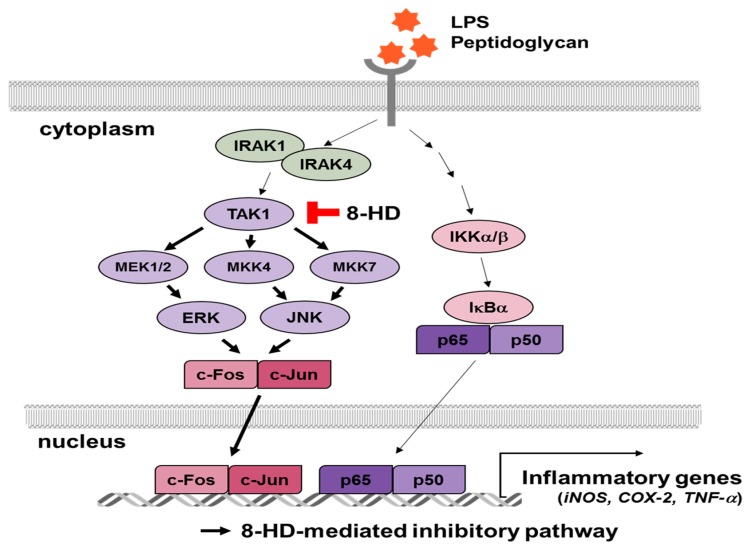
The regulatory effects of 8-HD on inflammatory signaling pathways. In the AP-1 signaling pathway, 8-HD suppresses the activity of TAK1, and subsequently, the transcriptional activity of c-Fos is also inhibited.

To confirm the inhibitory effect of 8-HD on TAK1, we transfected a TAK1 construct into HEK293T cells. We then screened for the downstream molecules MEK1/2, MKK4, and MKK7. Consistent with our previous results, the activities of MEK1/2, MKK4, and MKK7 were downregulated (Figure 5d). The immunoblotting results showed that 8-HD targets TAK1 and NF-κB. These findings imply that 8-HD regulates NF-κB and AP-1 signaling during TLR4-induced inflammatory responses (Figure 6).

## 3. Discussion

8-HD is known to have antioxidant, anti-proliferation, and depigmentation bioactivities [16,19,21]. Here, we further confirmed the antioxidant and anti-inflammatory effects of 8-HD. 8-HD suppresses the inflammatory responses triggered by different TLR ligands (Figure 3a). We explored the regulatory mechanisms of 8-HD, specifically in terms of the NF-κB and AP-1 inflammatory signaling pathways. 8-HD downregulates c-Fos transcriptional activity (Figure 4b,c). TAK1 was revealed as the target protein of 8-HD (Figure 5c,d).

The antioxidant effects of 8-HD were evaluated by various methods. Using the Fenton system, the xanthine oxidase system, and FRAP assays, G. Rimbach et al., and JS Park et al. observed that 8-HD scavenges hydroxyl radicals and superoxide, and reduces Fe^3+^ to Fe^2+^ [17,19]. We also confirmed the antioxidant effects of 8-HD using DPPH and ABTS assays. We observed that 8-HD significantly scavenges free radicals (Figure 2a,b). Moreover, we proved that 8-HD reduces cell-mediated radical production [17,19]. In fact, 8-HD has a potent ability to decrease various kinds of free radicals. It is worth noting that 8-HD is absorbed well during oral administration, showing antioxidant effects in vivo [18]. For these reasons, 8-HD has the potential to be used in drugs and health supplements.

8-HD suppresses NO production triggered by LPS (a TLR4 ligand), poly[I:C] (a TLR3 ligand), and PGN (a TLR2 ligand) (Figure 3b). In TLR4-induced inflammatory responses, 8-HD downregulates pro-inflammatory genes (e.g., iNOS, COX-2, and TNF-α) and modulates pro-inflammatory signaling pathways (Figure 3 and Figure 4). We speculate that 8-HD would regulate other TLR-induced inflammatory responses, given that there are known shared TLR signaling pathways [25,26]. The inhibitory effect of 8-HD on various TLR pathways could protect against the harmful side effects of inflammation during bacterial and viral infections.

We determined that 8-HD regulates TAK1 activity. Consequently, the transcriptional activities of the MAPKs and AP-1 are blocked (Figure 4c,d). In the case of NF-κB signaling, 8-HD inhibited the transcriptional activities of p65 and p50 (Figure 4b), but we did not observe a change in the phosphorylated forms of IκBα or IKKα/β (Figure 5a). In addition to the classical IKK/IκBα/NF-κB pathway, alternative pathways have been proposed—MAPK cascades could regulate the activation of NF-κB, or other kinases could phosphorylate the NF-κB subunits. ERK could act upstream of NF-κB, regulating its DNA-binding affinity. It is known that JNK and p38 are involved in cytoplasmic NF-κB activation and control its activity in the nucleus [27,28]. This suggests that 8-HD could regulate the transcriptional activation of NF-κB and AP-1 by inhibiting TAK1 and/or the MAPKs, as summarized in Figure 6. Apart from the IKKs, there are reports of other kinases that phosphorylate NF-κB. For example, p50 could be phosphorylated by protein kinase A (PKA) or Chk1, affecting its DNA-binding affinity. In the case of p65, GSK3β can phosphorylate the serine at position 468 [29]. The ability of 8-HD to inhibit the activities of these kinases should be addressed in future studies. Nonetheless, it is expected that the inhibitory activity of 8-HD on NF-κB activity is marginal, since the phosphorylation and degradation of IκBα are major pathways to activate NF-κB. Therefore, related points will be further studied to clarify this issue.

The industrial development of natural products is currently receiving much attention. Natural products have traditionally been a source of new drugs [30]. For example, eupatilin (sold as Stillen^®^, a 95% ethanol extract of *Artemisia asiatica* Nakai) is widely prescribed for gastritis and peptic ulcers in Korea [31,32]. In addition, the demand for natural and eco-friendly cosmetics is increasing [33,34]. Cosmetic ingredients with anti-inflammatory and antioxidant effects have been shown to reduce irritation [35,36]. Researchers have, in fact, tried to develop natural products for use across many industries [34,37,38]. In light of this trend, it is worth noting that 8-HD originates from soybeans. Like soybeans, 8-HD has potent antioxidant and anti-inflammatory effects. Used synthetically, 8-HD has the potential to be incorporated into many new products, including drugs, cosmetics, and health supplements. In addition, since our team has focused on its development as an immunomodulator targeted at skin inflammation, it is expected that this strategy will overcome various limits caused by liver metabolism and marginal in vivo absorption. Therefore, related studies regarding the pharmacokinetic and pharmacodynamics properties of 8-HD under topical and oral administration conditions will continuous investigate this possibility.

## 4. Materials and Methods

### 4.1. Biochemical Reagents

8-hydroxydaidzein (8-HD) was purchased from INDOFINE Chemical Company, Inc. (Hillsborough, NJ, USA). RAW264.7 cells (a BALB/c-derived murine macrophage cell line, No. TIB-71) and HEK293T cells (a human embryonic kidney cell line, No. CRL-3216) were acquired from the American Type Culture Collection (Rockville, MD, USA). 2,2-diphenyl-1-picrylhydrazyl (DPPH), 2,2′-azino-bis(3-ethylbenzothiazoline-6-sulphonic acid) diammonium salt (ABTS), ascorbic acid, NG-nitro-L-arginine methyl ester (L-NAME), polyethylenimine (PEI), lipopolysaccharide (LPS, *Escherichia coli* 0111:B4), and peptidoglycan (PGN) were obtained from Sigma Chemical Co. (St. Louis, MO, USA). Poly[I:C] was purchased from Calbiochem (La Jolla, CA, USA). 3-(4,5-dimethylthiazol-2-yl)-2,5-diphenyltetrazolium bromide (MTT) was purchased from Amresco (Solon, OH, USA). Fetal bovine serum (FBS) was purchased from Biotechnics Research (Lake Forest, CA, USA). RPMI1640 and DMEM were obtained from Hyclone (Grand Island, NY, USA). Antibodies against the total and phosphorylated forms of p65, p50, IκBα, IKKα/β, ERK, JNK, p38, MEK1/2, MKK4, MKK7, TAK1, HA, and β-actin were purchased from Cell Signaling (Beverly, MA, USA).

### 4.2. Cell Culture

RAW264.7 cells were cultured in RPMI1640 supplemented with 10% heat-inactivated FBS and 1% penicillin-streptomycin. HEK293T cells were cultured in DMEM with 5% heat-inactivated FBS and 1% penicillin-streptomycin. All cells were incubated at 37 °C in a 5% CO_2_ humidified incubator.

### 4.3. DPPH Assays

DPPH decolorimetric assays were performed to examine the scavenging effect of 8-HD [39,40]. A mixture of 8-HD (0–50 μM) and 250 μM DPPH was incubated at 37 °C for 30 min. Ascorbic acid (500 μM) was used as a positive control. After incubation, the absorbance at 517 nm of each sample was measured by spectrophotometry. The DPPH scavenging effect was expressed as the percent inhibition:

DPPH scavenging effect (%) = [(A_0_ − A_1_)/A_0_] × 100

where A_0_ is the absorbance of DPPH, and A_1_ is the absorbance of the sample.

### 4.4. ABTS Assays

ABTS scavenging assays were performed with modifications [41]. A mixture of 7.4 mM ABTS and 2.4 mM potassium persulfate (at a ratio of 1:1) was incubated at room temperature overnight to generate ABTS radical cations (ABTS•+). Solutions of 8-HD and ABTS were loaded into 96-well plates at a ratio of 1:1. Ascorbic acid (50 μM) was used as a positive control. After 30 min of incubation at 37 °C, the absorbance of each fraction was measured at 730 nm. The ABTS scavenging effect was expressed as a percentage:

ABTS scavenging effect (%) = [(A_0_ − A_1_)/A_0_] × 100

where A_0_ is the absorbance of ABTS, and A_1_ is the absorbance of the sample.

### 4.5. NO Production and Griess Assays

RAW264.7 cells (1 × 10^6^ cells/mL) were seeded in 96-well plates and incubated overnight. Cells were pre-treated with 8-HD (0–50 μM) or L-NAME (0–1000 μM) for 30 min, and then LPS (1 μg/mL) was added for 24 h. NO production was determined using Griess reagent, as previously reported [39,42].

### 4.6. Cell Viability Assays

RAW264.7 cells (1 × 10^6^ cells/mL), HEK293 cells (5 × 10^5^ cells/mL), and HaCaT cells (5 × 10^5^ cells/mL) were plated in 96-well plates. Cells were treated with 8-HD (0–50 μM) for 24 h, and MTT solution was then added for 3 h. Cytotoxicity was measured by conventional MTT assay.

### 4.7. Preparation of mRNA and Semi-Quantitative PCR

To quantify the expression of pro-inflammatory cytokines, RAW264.7 cells were pre-treated with 8-HD for 30 min. Cells were then exposed to LPS for 6 h. Total RNA was isolated with TRIzol reagent according to the manufacturer’s instructions. Semi-quantitative PCR was conducted as previously described [43].

### 4.8. Plasmid Transfections

HEK293T cells (3 × 10^5^ cells/mL) were seeded in 24-well plates. HA-TAK1 plasmids were transfected into HEK293T cells using PEI.

### 4.9. Preparation of Whole Cell Lysates and Immunoblotting

Cells were washed with PBS and collected. Cells were centrifuged at 12,000 rpm for 5 min at 4 °C. Cells were lysed with lysis buffer (20 mM Tris-HCl, pH 7.4; 2 mM ethyleneglycotetraacetic acid (EDTA); 2 mM ethyleneglycotetraacetic acid (EGTA); 1 mM DTT; 50 mM β-glycerol phosphate; 0.1 mM sodium vanadate; 1.6 mM pervanadate; 1% Triton X-100; 10% glycerol; 10 μg/mL aprotinin; 10 μg/mL pepstatin; 1 mM benzamide; and 2 mM PMSF). Protein lysates were pelleted by centrifugation (12,000 rpm, 5 min, 4 °C). Supernatants were used for Western blot analyses. The phosphorylated and total forms of p65, p50, IκBα, IKKα/β, ERK, JNK, p38, MEK1/2, MKK4, MKK7, TAK1, HA, and β-actin were used [44].

### 4.10. Statistical Analyses

The results were analyzed using an ANOVA/Scheffe’s post hoc test or the Kruskal–Wallis/Mann–Whitney tests. A *p*-value < 0.05 was considered statistically significant. All of the statistical tests were carried out using the computer program SPSS (SPSS Inc., Chicago, IL, USA).

## Abbreviations

8-HD	8-hydroxydaidzein
SNP	sodium nitropursside
ROS	radical oxygen species
iNOS	inducible nitric oxide synthase
COX	cycloocygenase
TNF	tumor necrosis factor
PAMPs	pattern-associated molecular patterns
PRRs	pattern-recognition receptors
NLRs	NOD-like receptors
RLRs	RIG-I-like receptors
TLRs	Toll-like receptors
MyD88	myeloid differentiation primary response 88
TRIF	TIR domain-containing adaptor-including interferon-β
IKK	IκB kinase
IL	interleukin
MAPKs	mitogen-activated protein kinases
ERK	extracellular signal-regulated kinase
JNK	c-Jun N-terminal kinase
MEK	MAPK/ERK kinase
MKK	mitogen-activated protein kinase kinase
TAK1	transforming growth factor beta-activated kinase 1
GSK	glycogen synthase kinase
ABTS	2,2′-azino-bis(3-ethylbenzothiazoline-6-sulphonic acid) diammonium salt
DPPH	2,2-diphenyl-1-picrylhydrazyl
LPS	lipopolysaccharide
poly[I:C]	polyinosinic-polycytidylic acid
PGN	peptidoglycan
NO	nitric oxide
L-NAME	NG-nitro-L-arginine methyl ester
PEI	polyethylenimine
MTT	3-(4,5-dimethylthiazol-2-yl)-2,5-diphenyltetrazolium bromide
FBS	fetal bovine serum
EDTA	ethylenediaminetetraacetic acid
EGTA	ethylene glycol-bis(β-aminoethyl ether)-N,N,N′,N′-tetraacetic acid
MDPI	Multidisciplinary Digital Publishing Institute
DOAJ	Directory of Open Access Journals
TLA	three-letter acronym
LD	linear dichroism
